# The Prevalence and Impact of Alzheimer’s and Related Dementia on Cirrhosis: A Retrospective Study

**DOI:** 10.1093/geroni/igaf122.2534

**Published:** 2025-12-31

**Authors:** Sijia Wei, Suyanpeng Zhang, Saad Hussain, Salva Balbale, Lihui Zhao, Nikhilesh Mazumder, Sanjay Mehrotra, Daniela Ladner

**Affiliations:** Northwestern University, Chicago, Illinois, United States; Northwestern University, Evanston, Illinois, United States; Northwestern University, Evanston, Illinois, United States; Northwestern University, Evanston, Illinois, United States; Northwestern University, Evanston, Illinois, United States; University of Michigan, Ann Arbor, Michigan, United States; Northwestern University, Evanston, Illinois, United States; Northwestern University Feinberg School of Medicine, Chicago, Illinois, United States

## Abstract

Alzheimer’s and related dementia (ADRD) complicates cirrhosis management; particularly, its symptoms overlap with hepatic encephalopathy. However, ADRD prevalence and impact on cirrhosis remain unclear. This study examined ADRD prevalence and its association with 1-year hospitalization and 5-year mortality in cirrhosis. Using electronic health records data, this retrospective study included 36,971 adults diagnosed with cirrhosis between 2011 and 2021 across seven health systems in a large US metropolitan area. ADRD diagnosis and certainty were determined using ICD-9/10 codes, medications, specialist involvement, and diagnostic timing. Multivariable logistic regressions and the Cox proportional hazards models assessed ADRD risk factors and influence on 1-year hospitalization and 5-year mortality. Among patients with cirrhosis, 5.7% (n = 2091) had an ADRD diagnosis: 2.0% (n = 727) before cirrhosis diagnosis, 2.3% (n = 844) after, and 1.5% potentially misdiagnosed hepatic encephalopathy. Compared to those without ADRD and after adjusting for covariates, ADRD before cirrhosis was associated with increased mortality in compensated (HR = 1.72, p < 0.001) but not in decompensated cirrhosis (HR = 0.89, p = 0.41). ADRD before cirrhosis increased the likelihood of 1-year hospitalization in both compensated (OR = 3.32, p < 0.001) and decompensated (OR = 1.58, p = 0.04). ADRD after cirrhosis was associated with lower mortality in both compensated (HR = 0.33, p < 0.001) and decompensated (HR = 0.41, p < 0.001) but was not significantly associated with hospitalization. Key factors associated with ADRD diagnosis and outcomes included age, metabolic-associated alcohol-associated liver disease, alcohol-associated liver disease, and being black American. Better detection and management of ADRD may improve cirrhosis outcomes, particularly in compensated cirrhosis. Further research is needed to understand the role of ADRD diagnosis timing in cirrhosis management.

